# Idiopathic Multicentric Castleman Disease Presenting With Hypertensive Choroidopathy: A Case Report

**DOI:** 10.7759/cureus.33368

**Published:** 2023-01-04

**Authors:** Tamami Mimoto, Noriyasu Hashida, Kohji Nishida

**Affiliations:** 1 Ophthalmology, Osaka University Graduate School of Medicine, Suita, JPN; 2 Ophthalmology, Osaka University, Suita, JPN

**Keywords:** case report, optical coherence tomography, malignant hypertension, choroid, castleman disease

## Abstract

Castleman disease (CD) is a lymphoproliferative disorder and rarely affects ocular tissue. This study aimed to report a case of hypertensive choroidopathy in a patient with Castleman’s disease associated with malignant hypertension. A 39-year-old man visited his local physician with fever, systemic edema, and multiple lymphadenopathies. An inguinal lymph node biopsy indicated CD. One month after the biopsy, the patient noted a blurring of vision. At the time of the initial examination at our hospital, his best-corrected visual acuity (BCVA) was 20/20 in both eyes but there were bilateral multiple Elschnig spots and sprinter hemorrhage at the fundus. Swept-source optical coherence tomography showed intra-retinal fluid, and serous retinal detachment (SRD). Fluorescein angiography revealed multiple punctate hyper fluorescences and indocyanine green angiography showed choroidopathy with increased vascular permeability. A general examination revealed symptoms of cardiac failure and multiple lymphadenopathies. Malignant hypertension with acute glomerulonephritis was diagnosed after a renal biopsy. After antihypertensive treatment, his blood pressure (BP) improved, and the SRD and choroidopathy promptly resolved. Presently, the patient is being followed up without complications. We report a case of hypertensive choroidopathy in a patient with CD associated with malignant hypertension. As a severe elevation in BP can damage choroidal vasculature and lead to vision loss, careful observation and active treatment are necessary.

## Introduction

Castleman disease (CD) is a lymphoproliferative disease characterized by hyperplasia and hypervascularization of lymphoid follicles throughout the body [1]. Clinically, the CD can be divided into unicentric CD (UCD) and multicentric CD (MCD). UCD is caused by lymph node involvement in a single regional area and may lack systemic symptoms. MCD is associated with multifocal lymph node enlargement and systemic inflammatory symptoms. MCD is classified into a hyaline-vascular type, plasma cell type, and mixed type based on histopathology [2-4]. The IL-6 produced by enlarged lymph nodes causes systemic inflammation, including fever, anemia, hypergammaglobulinemia, and elevated acute-phase proteins [2,5]. MCD can also be associated with human immunodeficiency virus (HIV) and human herpes virus 8 (HHV-8), and Epstein-Barr virus (EBV) infection must be investigated to exclude EBV-associated lymphoproliferative diseases [6]. Ocular involvement in CD is rare. Although several clinical manifestations, such as serous retinal detachment (SRD), choroidal infiltration [7-9], lacrimal gland enlargement, and hypertrophy of orbital soft tissues [10], have been reported, the detailed mechanism is not known. We report a rare case of hypertensive choroidopathy in a patient with CD associated with malignant hypertension.

## Case presentation

**Figure 1:**
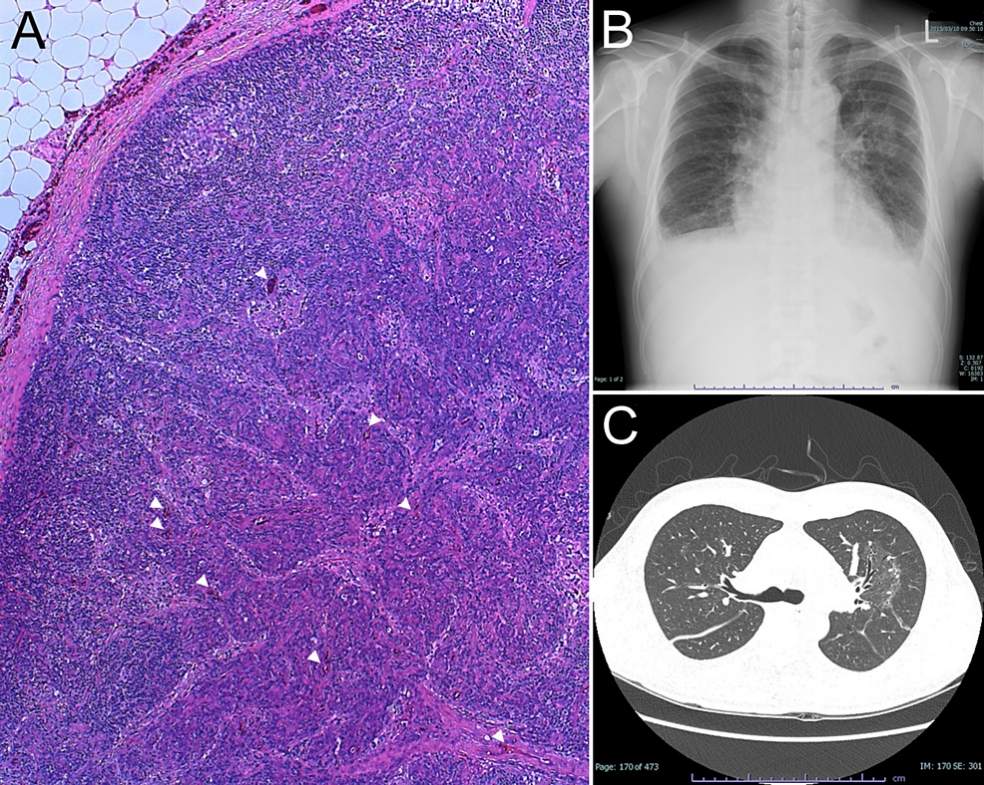
General examination at the first visit. (A) Histopathological findings of inguinal lymph node biopsy showing multiple lymphoid follicles and vascular invasion into the follicles (arrowheads). Lymphocytes are arranged in concentric circles surrounding the mantle layer outside the follicle, suggesting multicentric Castleman disease. (B) Thoracic x-ray photograph showing bilateral hilar lymphadenopathy and pleural effusion, infiltrative shadows, and cardiomegaly. (C) Chest computed tomography showing multiple lymph node enlargements in the mediastinum and hilar regions.

A 39-year-old man visited his local physician with fever, anasarca, and multiple (mediastinal, hilar, axillary, and inguinal lymph nodes) lymphadenopathies. Inguinal lymph node biopsy showed no findings suggestive of an aggressive hematopoietic tumor but findings consistent with CD (Figure 1A). One month after the biopsy, the patient complained of blurred vision and was referred to Osaka University Hospital. At the time of the initial examination, his blood pressure (BP) was 181/122 mmHg. A general examination revealed edema in the lower leg and obvious orthopnea. Blood examination showed mild anemia, and decreased renal function, with BUN 21 mg/dl, Cr 1.59 mg/dl, and eGFR 41.1 mL/min/1.80 m^2^, urine protein/creatinine ratio (UPr/UCr) 0.67 and an increased inflammatory response, with CRP 12.3 mg/dl, high immunoglobulin at IgG 2200 mg/dl, and γ-globulin 24.8%. Levels of serum interleukin 6 (IL-6) and vascular endothelial growth factor (VEGF) were 14 pg/mL (normal; <4.0 pg/mL) and 1650 pg/mL (normal; <38.3 pg/mL), respectively. The tumor marker soluble IL-2 receptor (sIL-2R) was also elevated (1130 pg/mL). The patient was negative for HIV, HHV 8, and EBV based on antibody titers. Immunoglobulin and T cell receptor (TCR) gene rearrangements were not detected. Chest x-ray showed bilateral pleural effusions and cardiomegaly (Figure 1B). A computed tomography scan of the chest also revealed ground-glass opacity and multiple lymphadenopathies (Figure 1C). These clinical manifestations are typical of MCD of the plasma cell type.

**Figure 2:**
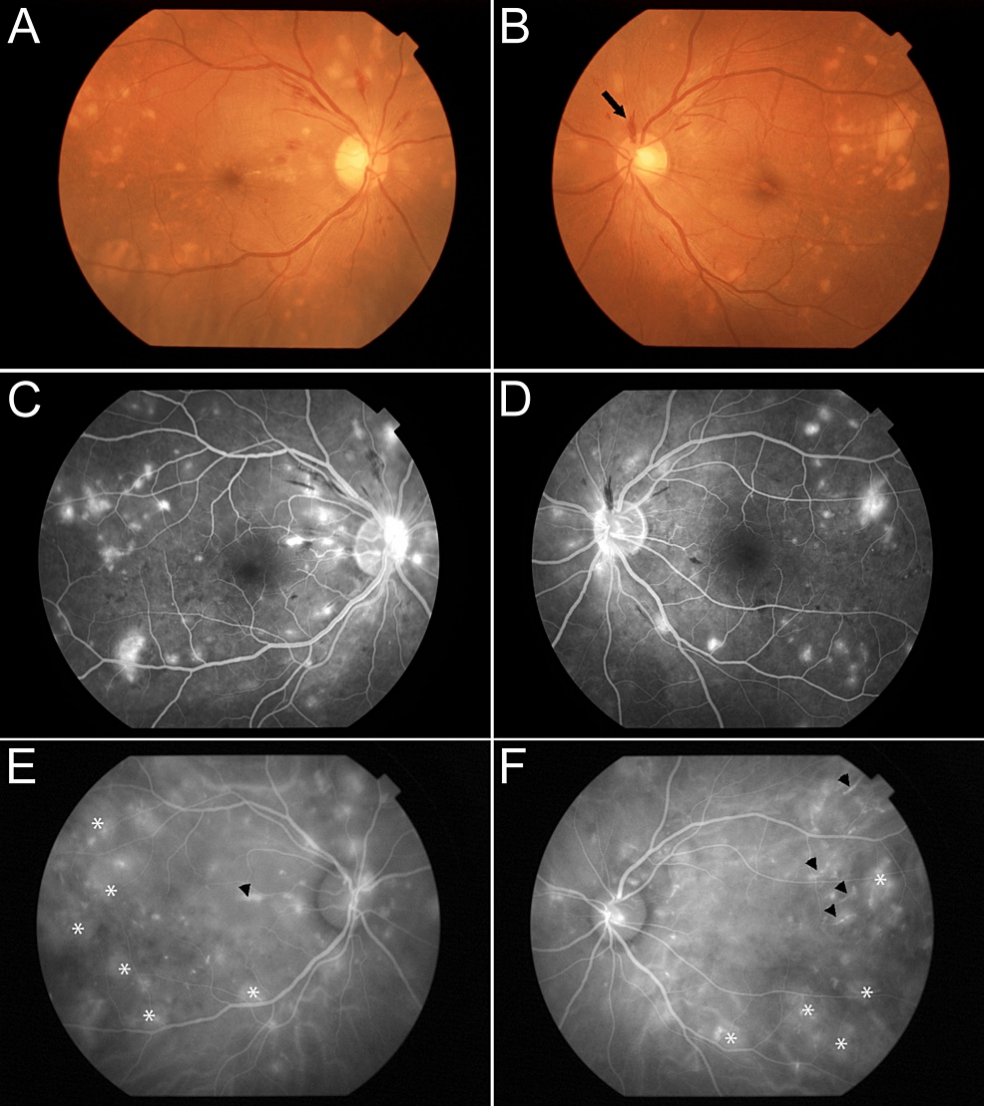
Ocular manifestation before treatment. (A, B) Fundus photograph shows multiple bilateral gray–yellow patches (Elschnig spots) and sprinter hemorrhage in the optic disc (arrow). Bilateral Narrowed arterioles and vascular tortuosity were observed. (C, D) Fluorescein angiography shows bilateral punctate hyperfluorescence, and staining was observed in line with the location of the Elschnig spot. (E, F) Indocyanine green angiography shows bilateral extensive mottled choroidal staining (*) and partial bright staining of choroidal vessels (arrowheads).

The best-corrected visual acuity (BCVA) of the patient was 20/20 in both eyes, and his intraocular pressure was normal. Anterior segment examination with a slit lamp was normal in both eyes. Fundus examination revealed multiple Elschnig spots (gray-yellow patches) at the level of the retinal pigment epithelium (RPE), and a sprinter hemorrhage was located around the optic disc (Figures 2A, 2B).

**Figure 3:**
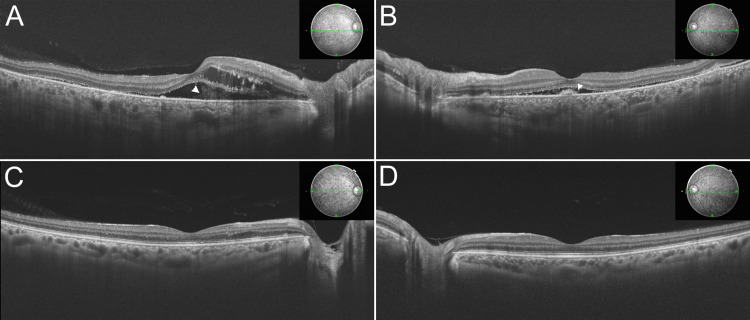
Optical coherence tomography findings before and after treatment. (A), (B) Optical coherence tomography showing exudative retinal detachment involving the fovea. Bilateral fibrin formation was observed in the subretinal space (arrowheads). (C), (D) Recovery of the retinal and choroidal structures. An increase in choroidal thickness was observed in the acute phase and recovered with treatment.

Swept-source optical coherence tomography (SS-OCT) examination showed intraretinal fluid in the right eye, bilateral serous retinal detachment (SRD), and exudates on RPE corresponding to Elschnig spots (Figures 3A, 3B). Fluorescein angiography (FA) showed multiple punctate hyperfluorescence and staining in line with the location of the Elschnig spot (Figures 2C, 2D). According to indocyanine green angiography (IA), scattered tissue staining and bright choroidal vessel staining, and increased choroidal vascular permeability were present (Figures 2E, 2F).

**Figure 4:**
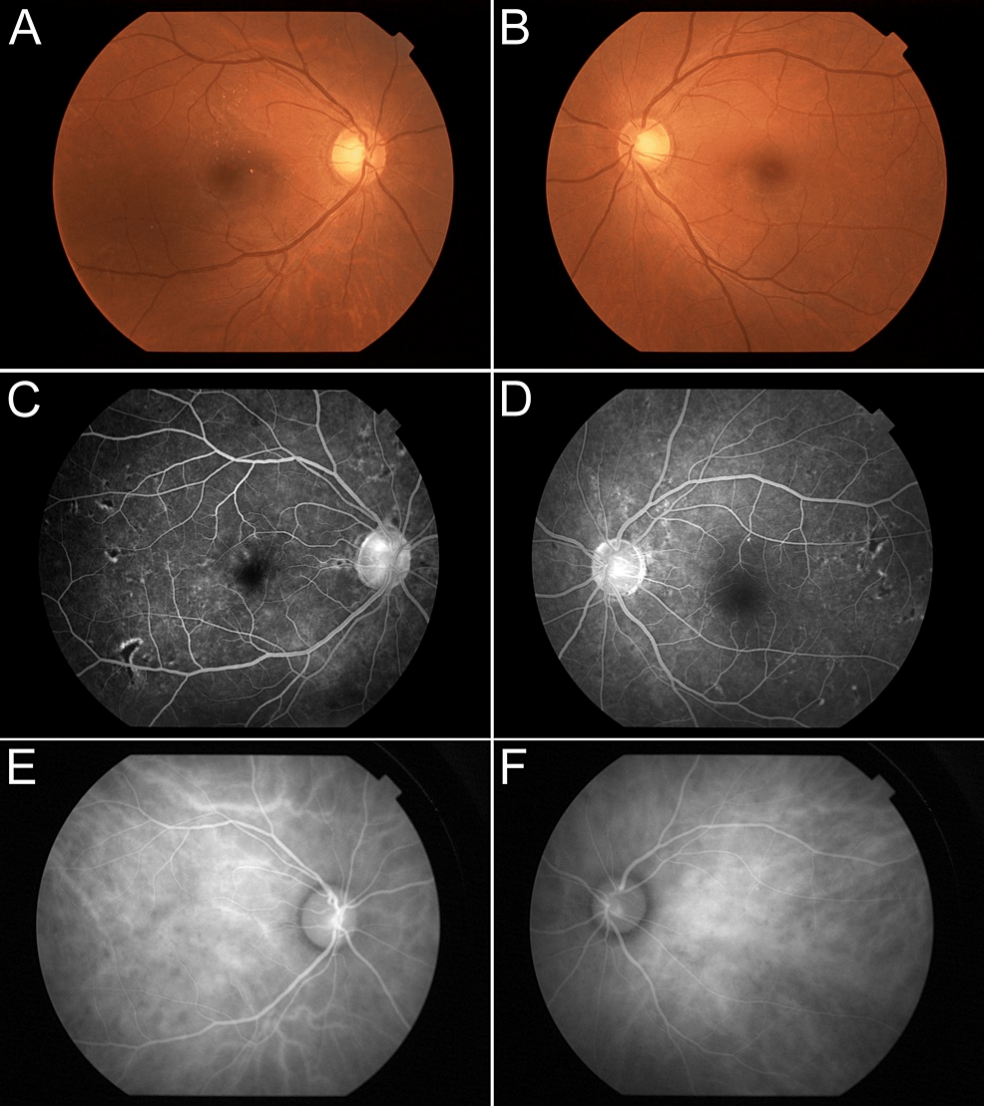
Ocular manifestation after treatment. (A), (B) Fundus photograph revealed that the bilateral Elschnig spots and hemorrhage disappeared. (C), (D) Fluorescein angiography shows scattered window defects and loss of staining. (E), (F) Indocyanine green angiography shows that choroidopathy with increased vascular permeability disappeared, resulting in normal contrast findings.

Ten days after the first visit, the systemic evaluation revealed that his systolic blood pressure remained at 200 mmHg. He was admitted to the nephrology clinic at the time of the diagnosis of heart failure, and antihypertensive and diuretic medication was started. A renal biopsy performed during hospitalization was consistent with malignant hypertension with acute glomerulonephritis. Narrowing of the vascular lumen and atrophy of the glomerulus were observed. For the management of hypertension, we used short-lived, and rapid-acting intravenous hypotensive agents: nicardipine and angiotensin II Receptor Blocker: Benicar. One week later, his systolic blood pressure improved to the 120-130 mmHg range. His SRD and retinal edema gradually resolved within a matter of two weeks (Figures 3C, 3D), and the fundus appearance normalized (Figures 4A, 4B). FA examination showed that the filling defect remained partial but that both hyperfluorescence and blocked fluorescence resolved (Figures 4C, 4D). Choroidal vascular hyperpermeability was normalized (Figures 4E, 4F).

At three months after the start of treatment, SRD and hyperreflective material under the retinal pigment epithelium disappeared in both eyes (Figures 3C, 3D). His visual acuity improved to 20/20 in the right eye and 20/15 in the left eye. Presently, the patient is being followed up without complications. The subsequent clinical course was uneventful.

## Discussion

CD rarely leads to direct involvement of the orbit or ocular structures due to a lack of lymphoid tissue in the eye [10]. Several investigators have reported rare intraocular involvement [7-12]; however, the pathogenic mechanism of ocular involvement is not fully understood. We report a case of hypertensive choroidopathy in a patient with CD associated with malignant hypertension. Although CD can be associated with hypertension, there are few reports of severe choroidopathy resulting in vision loss [13,14]. The choroidopathy seen in this case was thought to be caused by rapid changes in blood pressure due to malignant hypertension. Emerson et al. reported choroidal invasion in a patient with CD [9]. The yellow exudative lesion on the RPE in this case was initially suspected to be caused by direct infiltration of the choroid associated with CD [2,9,15]. Based on OCT findings, it was determined that the exudative lesion was not in the choroid and that the integrity of the RPE was damaged, as previously reported [15,16].

In general, blood pressure elevations may affect the choroidal circulation [13,14], causing hypertensive choroidopathy, including Elschnig spots and Siegrist streaks [16-18]. However, the changes in retinal vessels such as arteriolar narrowing usually seen in hypertensive patients were not prominent in our case; rather, choroidal changes were strong. The multiple yellowish lesions observed on the RPE were presumed to be Elschnig spots [16], which are thought to be RPE changes associated with hypertension [17]. In addition, the contrast staining of some vessels in IA was thought to be the result of damage to the fibrinoid-denuded choroidal vessel wall due to the rapid increase in blood pressure resulting from malignant hypertension [18,19]. Malignant hypertension is a condition in which severe elevation of blood pressure causes acute damage to target organs such as the brain, heart, and kidneys, and it requires prompt and appropriate diagnosis and immediate antihypertensive treatment [20]. In this case, the choroid and the kidney were mainly affected because they are endo-organs [13]. According to previous reports [13,14], CD can be associated with hypertension. Therefore, lack of therapeutic intervention may have led to malignant hypertension and choroidopathy. The choriocapillaris in the choroid and the glomerular vasculature in the kidneys are important for organ function [14,18,20]. The rapid pressure change caused by malignant hypertension may have caused damage to these organs, which have fine vascular structures [18-20].

## Conclusions

We report a rare case of a multicentric CD patient who showed malignant hypertension with acute glomerulonephritis and hypertensive choroidopathy that eventually resolved with antihypertensive treatment. A sudden change in blood pressure may lead to retinal choroidal lesions. Although the detailed mechanism of the uncommon clinical course and rare histopathological pattern are unclear, periodic evaluation of intraocular lesions and active systemic treatment is very important.
